# Mitochondrial NME6 Influences Basic Cellular Processes in Tumor Cells In Vitro

**DOI:** 10.3390/ijms25179580

**Published:** 2024-09-04

**Authors:** Bastien Proust, Anđela Horvat, Ana Tadijan, Ignacija Vlašić, Maja Herak Bosnar

**Affiliations:** Division of Molecular Medicine, Ruđer Bošković Institute, Bijenička Cesta 54, 10002 Zagreb, Croatia; bproust@irb.hr (B.P.); arunje@irb.hr (A.H.); ana.tadijan@irb.hr (A.T.); ivlasic@irb.hr (I.V.)

**Keywords:** NME6, mitochondria, migration potential, cell cycle, proliferation, apoptosis

## Abstract

NME6 belongs to the family of nucleoside diphosphate kinase enzymes, whose major role is to transfer the terminal phosphate from NTPs, mostly ATP, to other (d)NDPs via a high-energy intermediate. Beside this basic enzymatic activity, the family, comprising 10 genes/proteins in humans, executes a number of diverse biochemical/biological functions in the cell. A few previous studies have reported that NME6 resides in the mitochondria and influences oxidative phosphorylation while interacting with RCC1L, a GTPase involved in mitochondrial ribosome assembly and translation. Considering the multifunctional role of NME family members, the goal of the present study was to assess the influence of the overexpression or silencing of NME6 on fundamental cellular events of MDA-MB-231T metastatic breast cancer cells. Using flow cytometry, Western blotting, and a wound-healing assay, we demonstrated that the overexpression of NME6 reduces cell migration and alters the expression of EMT (epithelial–mesenchymal transition) markers. In addition, NME6 overexpression influences cell cycle distribution exclusively upon DNA damage and impacts the MAPK/ERK signaling pathway, while it has no effect on apoptosis. To conclude, our results demonstrate that NME6 is involved in different cellular processes, providing a solid basis for future, more precise investigations of its role.

## 1. Introduction

Nucleoside diphosphate kinases (NDPK/NME/Nm23) form a large family of enzymes whose major function is the transfer of the terminal phosphate group from NTPs to (d)NDPs through a high-energy phospho-histidine intermediate, thus maintaining the cellular NTP pool [1]. Although initially discovered as exclusively “house-keeping” enzymes, over several decades of intensive research, it has become clear that NME proteins are multifunctional, with a plethora of biochemical and biological functions in different cellular compartments.

The human NME family consists of 10 genes divided into two groups according to their phylogenetical analysis and gene/protein structure [2]. Group I (NME1–NME4) are highly homologous in gene/protein sequence. They emerged from a single ancestral gene on the level of vertebrates [3]. They possess the nine amino acid residues critical for protein stability and activity and display only one NDPK domain. They are proven to possess the NDP kinase activity in their hexameric form [1]. Most of the cellular NDPK activity is due to NDPKA/NME1 and NDPKB/NME2 which combine to form different homo- or heterohexameric isoenzymes (A6… A5B, B6) [4]. Interest in these enzymes increased significantly after the discovery of Steeg et al. [5], which showed that NME1 is able to suppress one or more steps in the metastatic cascade. Therefore, NME1 was appointed the first of many (more than 30) metastasis suppressor genes/proteins discovered in the last three decades [6]. NME1 and NME2 are highly homologous (with 88% amino acid identity) and take part in numerous basic cellular processes [7,8,9,10,11,12,13,14,15,16,17]. The two remaining Group I members, NME3 and NME4, reside in the mitochondria [18,19], while NME4 is the only NME protein that has a canonical mitochondrial targeting sequence. The more versatile Group II (NME5–NME9) members emerged earlier in the course of evolution and show less homology among themselves (28–45% amino acid identity) as well as in comparison to homologs in different species (25–34% amino acid identity) [20]. Group II NMEs display one or more NDPK domains, and it is still unclear whether they possess NDPK activity. Since NME1 and NME2 were connected to metastasis formation immediately after their discovery, little attention was dedicated to the Group II family members for decades afterwards. However, this trend started to change, especially concerning NME6.

NME6 has simultaneously been discovered by two separate groups. Mehus and coworkers [21] identified NME6 on the chromosome location 3p21.3. They predicted a protein of 186 amino acids with a calculated pI of 8.5 and a molecular weight of 21,142 Da. Compared to the extensively investigated NME1, NME6 contains seven additional residues at its N-terminus, one additional amino acid at position L-130, 22 on its C-terminus, and three additional residues in the *Kpn* (killer of the prune) loop [22]. Prior to the C-terminus, a cluster of four E (glutamic acid) can be found. Despite having a relatively low level of homology with other NME proteins [2,8,23,24], NME6 contains all the residues necessary for nucleotide binding and catalysis, which encompass the amino acid residues necessary for obtaining a hexameric structure. They also reported NME6 to be expressed at a moderate level (lower than NME1/2) in many human tissues such as kidney, prostate, ovary, spleen, and intestine. The authors failed to produce a recombinant protein in a bacterial system [21]. Tsuiki and coworkers [25] demonstrated that NME6 colocalizes, at least partly, in mitochondria. The protein they produced displayed lower NDPK activity compared to NME1/2. The overexpression of NME6 in SAOS2 (human osteosarcoma) cells resulted in growth suppression and the formation of multinucleated cells after 72 h; therefore, they concluded that NME6 probably affects cytokinesis [25]. Our recent work added substantially to the pioneering work described so far and resolved several discrepancies [26]. In brief, using mass spectrometry, we demonstrated that human NME6 occurs in cells as two isoforms (194 aa and 186 aa), with the shorter one being predominantly represented. Despite the absence of a canonical mitochondrial targeting sequence (MTS), we localized NME6 in the mitochondrial matrix, adjacent to the mitochondrial inner membrane (MIM). NME6 prefers the monomeric structure and, as a consequence, does not exhibit NDP kinase activity in vitro. Our screening for potential protein partners revealed an association with NME4 and OPA1 (OPA1 mitochondrial dynamin like GTPase), but a direct, physical interaction was confirmed only with RCC1L (RCC1-like G exchanging factor-like protein (WBSCR16)), a protein involved in mitochondrial ribosome assembly and mitochondrial translation. Moreover, the overexpression of NME6 reduced ADP-stimulated mitochondrial respiration, linked to a downregulation of respiratory complexes’ expression (CIII, CIV, and CV). Furthermore, recent studies have confirmed the NME6/RCC1L interaction, but also identified NME6 as an essential protein in supplying mitochondrial ribonucleotides. These studies have shown, albeit indirectly, that NME6 can display NDP kinase activity exclusively through an obligatory interaction with RCC1L and, thus, highlighted the importance of NME6 for maintaining the mitochondrial pyrimidine nucleotide pool [27,28].

Although NME6 has recently been intensively studied at the protein level, little is known about its impact on basic cellular events. Only a few studies have linked NME6 to various pathologies and disease mechanisms. NME6 was found to be overexpressed along with NME4 and NME7 in primary colon and gastric carcinomas [29], while another study revealed that NME6 expression was higher in colorectal cancer tissues [30]. In an shRNA screening, NME6 and NME7 were found to be essential for embryonic stem cell renewal [31]. More recently, the potential role of NME6 in the regulation of inflammation in mice has been described [32].

Given the relevance of several NME proteins in fundamental cellular processes and the regulation of metastasis spreading, as well as the presence of NME6 within mitochondria influencing de facto cellular energy production, we aimed to study the impact of NME6 on basic cellular functions. Therefore, proliferation, cell cycle progression, apoptosis, and metastatic potential were investigated after NME6 overexpression or silencing in breast cancer cells. Our results show that NME6 has no impact on apoptosis or the cell cycle under normal conditions. However, NME6 overexpression increases the number of 4N cells after DNA damage induced by etoposide. Moreover, our study revealed that the levels of both cell cycle progression marker cyclin A and the proliferation marker PCNA (proliferating cell nuclear antigen) decrease upon NME6 overexpression. In addition, increased NME6 levels moderately hinder the activity of the MAPK/ERK signaling pathway. To the best of our knowledge, our study is the first that has shown that NME6 overexpression strongly decreases the migration capacity of metastatic breast cancer cells and changes the expression levels of characteristic EMT markers. Based on our understanding of the NME field, we can assume that all the observed changes are due to the influence of NME6 on energy levels, at least in our cell line models.

## 2. Results and Discussion

NME6 was shown to be associated with mitochondrial proteins and negatively regulate ADP-stimulated respiration (OXPHOS) [26], thereby potentially influencing mitochondrial functions that can contribute to cancer cell hallmarks [33]. To explore this eventuality in more detail, we analyzed basic cellular processes such as cell cycle progression, cell proliferation, migration potential, and apoptosis, as well as the expression of relevant markers associated with these cellular processes after NME6 knock-down or overexpression in cancer cell lines. In our previous work, we showed that the 186 aa isoform of NME6 is predominantly expressed in various cancerous and non-cancerous cells [26]; therefore, in this study, “NME6” refers to the isoform of 186 amino acids. In addition, we have produced stable monoclonal MDA-MB-231T cell lines either overexpressing NME6 (KI-NME6) or “empty” vectors containing clones (KI-CTRL). The molecular weight difference between the endogenous NME6 and overexpressed NME6 is expected, due to the presence of FLAG-tag on the exogenous NME6 protein. As we were unable to produce stable knock-out clones, the cells were transfected either with NME6 silencers (Si-NME6) or scramble siRNA as a negative control (Si-CTRL) [26]. The same material was used in the present study.

### 2.1. NME6 Overexpression Influences Cell Cycle Progression after DNA Damage and Changes the Level of Cell Cycle Regulators

The potential impact of NME6 on cell cycle regulation in MDA-MB-231T cells was assessed by analyzing the cell cycle distribution using flow cytometry, measuring the fluorescence intensity of DNA-intercalated propidium iodide as described previously [34].

**Figure 1:**
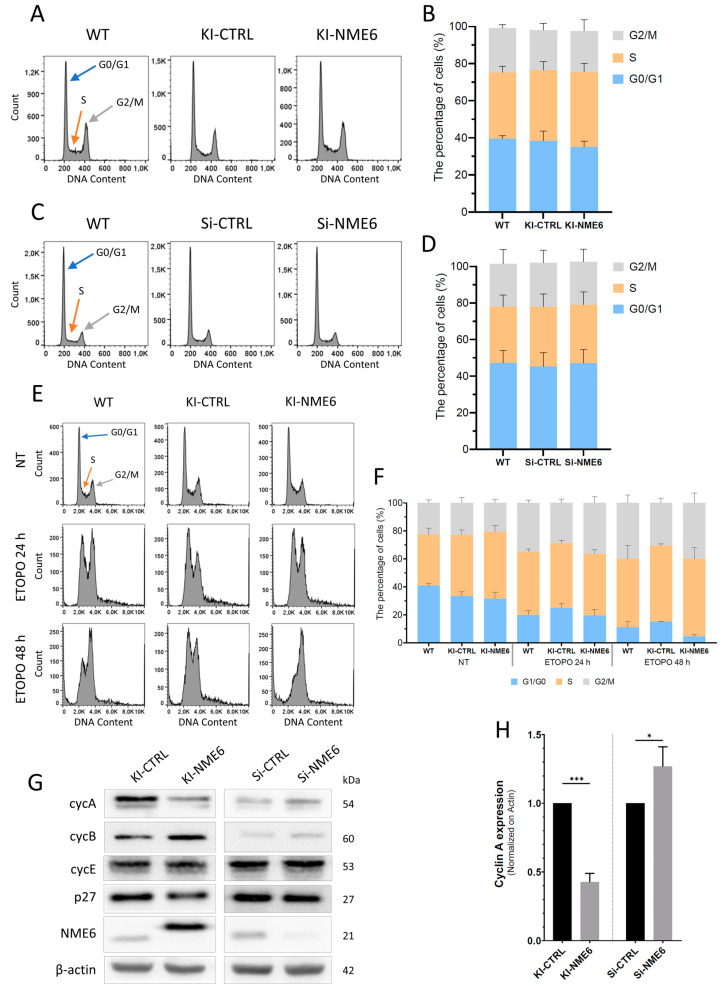
NME6 overexpression influences cell cycle profile after DNA damage and the level of cell cycle regulators. Analysis of MDA-MB-231T cell cycle distribution by flow cytometry upon NME6 overexpression (**A**,**B**) or silencing (**C**,**D**), as well as after 24 h and 48 h exposure to the DNA damaging agent etoposide (**E**,**F**). Levels of cell cycle regulators upon NME6 overexpression or silencing in MDA-MB-231T cells under normal conditions were analyzed by Western blotting. Molecular weight of every protein is annotated on the right side (**G**), while the corresponding blot densitometry test was performed for cyclin A (**H**). Significance is shown as *** *p* < 0.001; * *p* < 0.05 (unpaired *t*-test; *n* = 3).

No significant change was observed in cell cycle distribution between KI-NME6 and KI-CTRL or parental MDA-MB-231T cells (WT) (Figure 1A,B). Similarly, the silencing of NME6 in MDA-MB-231T cells (Si-NME6) had no effect on the cell cycle distribution when compared to a control (Si-CTRL) (Figure 1C,D). Therefore, the tuning of NME6 expression had no influence on the cell cycle distribution in MDA-MB-231T cells under normal conditions. These results are not in accordance with the previous findings of Tsuiki et al., which showed that the enhanced expression of the longer human NME6 isoform leads to an increase in the proportion of cells with a DNA content of 4N and polyploidy in the SAOS2 human osteosarcoma cell line [25]. However, those results were achieved by overexpressing NME6 using an inducible Cre-recombinase system in SAOS2 cells, whereas our experiments were performed on MDA-MB-231T clones stably overexpressing NME6. In addition, SAOS-2 cells lack the expression of p53 and, thus, are deficient in a pivotal tumor suppressor that is important for cell cycle regulation [35], whereas MDA-MB-231T cells express a mutant form of p53 [36]. In our cellular model, the constitutive overexpression of NME6 causes no long-term changes in cell cycle phase distribution under normal conditions (i.e., in the absence of any additional stress factor). As we did not observe significant differences in the cell cycle distribution profiles of asynchronously growing (non-treated) cells, we decided to explore the potential effect of NME6 overexpression on cell cycle regulation after DNA damage. For that purpose, we treated the cells with etoposide, a topoisomerase II inhibitor which causes G2/M arrest (Figure 1E,F). A decreased percentage of 2N cells and an increased level of 4N cells in KI-NME6 clones were observed compared to KI-CTRL and WT cells, indicating the potential involvement of NME6 protein in cell cycle regulation after DNA damage.

Cell cycle progression is controlled by cyclins and cyclin-dependent kinases (Cdks). Therefore, the possible changes in cell cycle progression associated with NME6 overexpression or silencing were further investigated by determining the expression profile of cyclins by Western blotting under normal conditions, i.e., without introducing DNA damage (Figure 1G). Upon NME6 overexpression, a drop in the level of cyclin A was observed (KI-NME6) compared to control clones (KI-CTRL). Inversely, NME6 silencing in parental MDA-MB-231T cells (Si-NME6) slightly increased the levels of cyclin A as compared to the control (Si-CTRL) (Figure 1G,H). The influence of NME6 on the abundance of cyclin A could imply the involvement of NME6 in the regulation of cell cycle progression. On the other hand, the levels of other cell cycle regulators upon NME6 up- or downregulation, such as cyclin B and cyclin E as well as p27, were not significantly affected (Figure 1G and Figure S1).

The results we obtained do not allow a straightforward conclusion to be drawn regarding the effect of NME6 on cell cycle progression. It could be hypothesized that the observed mild effect is due to the imbalance of energy caused by our tuning of NME6 expression. The complex processes of cell cycle progression require a considerable amount of energy [37], and there is a lot of evidence on the regulatory role of mitochondria in cell cycle control and vice versa. Cyclin D1 was shown to negatively regulate mitochondrial activity in breast cancer cells [38], Cdk1/cyclin B1 can promote mitochondrial fission by phosphorylating Drp1 [39], and a hyperfused mitochondrial state regulates cyclin E levels and the G1/S transition [40]. In addition, Cdk1/cyclin B1 can localize to the mitochondrial matrix and mediate the phosphorylation of OXPHOS CI subunits, thereby enhancing their activity and consequently increasing mitochondrial respiration, which ensures the cells have enough bioenergy for the G2/M transition [41,42]. Nevertheless, the precise molecular mechanisms underlying the regulation of mitochondrial respiration along cell cycle progression are still unknown. Future studies are needed to elucidate the link between NME6, a potential regulator of mitochondrial respiration [26,27], and cell cycle regulators such as cyclin A.

### 2.2. NME6 Overexpression Reduces Levels of PCNA Proliferation Marker and Moderately Affects MAPK/ERK Pathway Activity

**Figure 2:**
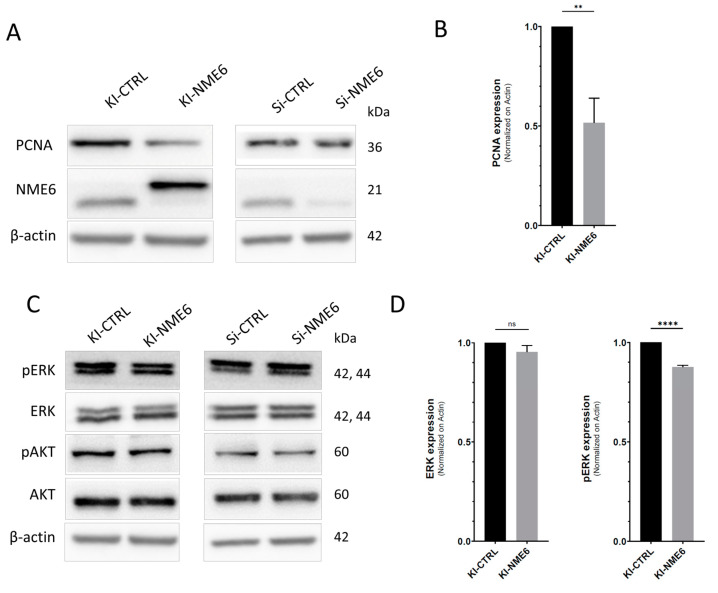
NME6 overexpression negatively impacts proliferation and MAPK/ERK pathway. The protein expression of PCNA proliferation marker (**A**) as well as the expression and phosphorylation status of PI3K/AKT and MAPK/ERK signaling pathways (**C**) was analyzed by Western blotting upon NME6 knock-in (KI-NME6) or silencing (Si-NME6) in MDA-MB-231T cells. Molecular weights of proteins are annotated on the right side of the membrane. The abundance of PCNA, ERK, and pERK proteins was analyzed by blot densitometry (**B**,**D**). Significance is shown as **** *p* < 0.0001; ** *p* < 0.01; ns: not significant (unpaired *t*-test; *n* = 3).

The negative effect of NME6 overexpression on cell growth has been reported in SAOS2 cells [25]. Since cyclin A is considered to be a good proliferation marker of breast cancer cells [43,44], and its level is influenced by NME6 in MDA-MB-231T breast cancer cells, we decided to examine the role of NME6 on proliferation. We assessed the effect of NME6 overexpression or silencing on the PCNA proliferation marker levels by Western blotting. The PCNA levels were reduced in KI-NME6 when compared to control cells (KI-CTRL) (Figure 2A,B). On the other hand, there was no difference in the PCNA levels after NME6 silencing (Figure 2A and Figure S2). The obtained results suggest the regulatory role of NME6 in cell growth and cell cycle progression, which is in line with the already published results of Tsuiki et al. [25]. Interestingly, several studies have shown that another member of the NME family, the mitochondrial protein NME4, also influences the proliferation of both non-small cell lung cancer cells and esophageal squamous cell carcinoma cells [45,46]. In more detail, NME4 silencing suppresses the proliferation of cancer cells and inactivates the STAT3 signaling pathway in squamous cell carcinoma cells [47], which implies that mitochondrial NME proteins can affect proliferation and potentially regulate the activity of different signaling pathways.

Many protein members of the PI3K/AKT or MAPK/ERK signaling pathways are known to be direct or indirect interacting partners of the PCNA proliferation marker [48]. Furthermore, both signaling pathways promote breast cancer cell growth and proliferation and thus foster cancer development and progression [49,50,51]. Therefore, to test the hypothesis that NME6 influences cancer cell growth and proliferation as well as signaling pathway activities, we checked whether NME6 status perturbs PI3K/AKT and/or MAPK/ERK signaling in MDA-MB-231T cells (Figure 2C). The activity of the PI3K/AKT or MAPK/ERK pathways was assessed by determining the ratio of phosphorylated and total ERK1/2 or AKT levels, respectively. A stable clone overexpressing NME6 (KI-NME6) showed reduced levels of phosphorylated ERK1/2 compared to the control (KI-CTRL) (Figure 2C,D), while the levels of phosphorylated AKT remained unchanged. Furthermore, minor or no changes in levels of phosphorylated AKT or phosphorylated ERK1/2, respectively, were observed upon NME6 silencing (Si-NME6) compared to the control (Si-CTRL) (Figure 2C and Figure S2). From our results, we conclude that although NME6 does not affect PI3K/AKT activity, it moderately affects the MAPK/ERK pathway.

### 2.3. NME6 Overexpression Decreases Migration and Alters the Expression of EMT Markers

Both the PI3K/AKT and MAPK/ERK signaling pathways can contribute to the EMT [52,53], which drives different cellular processes including cell migration, thus contributing to the metastatic potential of cancer cells. A recent study of Lacombe et al. shows that the mitochondrial protein NME4 exhibits metastasis suppressor properties which emphasize that other mitochondrial proteins could also have a role in regulating metastatic spread [54].

**Figure 3:**
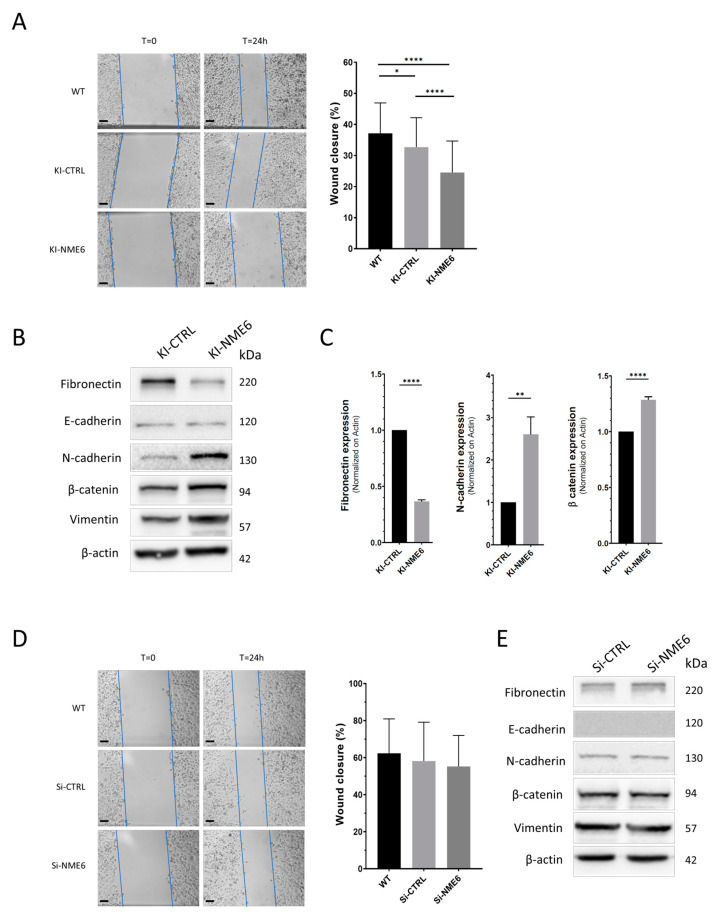
The overexpression of NME6 decreases cells’ migration potential. (**A**) Representative results of wound-healing assay of MDA-MB-231T cell line (WT), stable monoclonal cell line carrying empty vector as a negative control for clones (KI-CTRL), or stable monoclonal cell line overexpressing NME6 (KI-NME6) and associated statistical analysis (one-way ANOVA; *n* = 58, 95, and 103, respectively). Significance is given as **** *p* < 0.0001; * *p* < 0.05. (**B**) Western blot of EMT markers upon NME6 overexpression with molecular weights of proteins annotated on the right side of the membrane. The abundance of fibronectin, N-cadherin, and β-catenin proteins was analyzed by blot densitometry (**C**). Significance is shown as **** *p* < 0.0001; ** *p* < 0.01 (unpaired *t*-test; *n* = 3). (**D**) Representative results of the wound-healing assay of MDA-MB-231T (WT) cells transfected with scramble siRNA (Si-CTRL) or with NME6 silencers (Si-NME6) and associated statistical analysis (one-way ANOVA; *n* = 40, 39, and 40, respectively). (**E**) Western blot of EMT markers upon NME6 silencing with molecular weights of proteins annotated on the right side of the membrane. All scale bars: 200 µm.

To test the possible influence of NME6 on the migratory potential, we determined the effect of NME6 overexpression and silencing on MDA-MB-231T cell migration using the well-established wound-healing assay. In our experiments, the cells underwent serum starvation prior the creation of the wound and while the wound healed in order to minimize cell proliferation, which could interfere with the cell migration measurements [55]. The control clone (KI-CTRL) exhibited a slight decrease in migration potential compared to the WT control but with a weak statistical confidence (33% and 37% wound closure, respectively). However, the wound closure of the NME6-overexpressing clone (KI-NME6) was strongly decreased (25% wound closure), accompanied with a high statistical significance when compared to both controls (KI-CTRL and WT) (Figure 3A). It appears that the overexpression of NME6 strongly decreased the migratory potential of the MDA-MB-231T cells. On the other hand, the silencing of NME6 had no statistical effect on migration (Figure 3D). However, this result must be interpreted carefully, since the silencing of NME6 in our study does not totally suppress NME6 expression, but rather leads to a 70–80% decrease in protein expression. Therefore, we can assume that the remaining NME6 protein is sufficient to perform its cellular function, i.e., maintain the migration profile. It would be interesting to perform the wound-healing assay on stable monoclonal NME6 knockout (KO) cells, which we were unfortunately unable to produce [26].

NME6 is a mitochondrial protein that was shown to have a negative impact on mitochondrial respiration [26], and its overexpression could potentially deregulate energy production in mitochondria [27,28]. As cell migration consequently requires energy [56], the hypothesis of an energy shortage induced by the overexpression of NME6 could be a tempting way to explain the reduced migratory potential of MDA-MB-231T breast cancer cells. However, testing this hypothesis is beyond the scope of this work. Interestingly, Lacombe et al. detected a reduced migration ability of MDA-MB-231 cells upon the overexpression of NME4, which was shown to be negatively associated with the EMT process via the upregulation of epithelial markers and the downregulation of mesenchymal markers [54]. Based on our findings on the anti-migratory potential of NME6 in MDA-MB-231T cells, we hypothesized that NME6 could also be associated with the EMT process. The EMT is a process characterized by the transition of cancer cells from the epithelial to mesenchymal phenotype, which contributes to the increased migration capability of cancer cells [57]. The common feature of the EMT is the loss of E-cadherin and upregulation of mesenchymal markers, such as N-cadherin [57]. However, due to cancer cell plasticity, epithelial cells can exhibit a hybrid epithelial/mesenchymal phenotype and undergo a partial EMT associated with the advanced metastatic/tumorigenic features of cancer cells [58]. To test whether NME6 contributes to EMT-like cell features, we checked the expression of E-cadherin and different mesenchymal markers, e.g., fibronectin, N-cadherin, β-catenin, and vimentin upon NME6 overexpression or silencing (Figure 3B,E). After the NME6 overexpression (KI-NME6), we observed a significantly reduced expression of fibronectin and increased levels of both N-cadherin and β-catenin compared to the control (KI-CTRL) (Figure 3B,C). On the other hand, only slight or no change was observed in the expression levels of EMT markers upon NME6 silencing (Figure 3E and Figure S3). Based on our results, we can assume that only the increased levels of NME6 stimulate molecular changes associated with the (partial) EMT process.

### 2.4. NME6 Is Not Associated with Apoptosis but Moderately Affects the Level of p53 Family Members in Unstressed Conditions

NME6 is localized within the mitochondria, which represents an important hub in apoptosis. This led us to investigate its involvement in apoptosis processes. The possible impact of NME6 overexpression or silencing on apoptosis was studied using flow cytometry after staining with annexin V/propidium iodide. It is known that hotspot mutations located in the *TP53* exon region produce a mutant p53 protein form, which can negatively influence apoptosis [59]. Therefore, based on their different p53 status, RKO cells were chosen over MDA-MB-231T for this particular experiment. We, thus, measured the apoptosis status of RKO cells transiently overexpressing NME6 (OV-NME6) or NME6 silenced (Si-NME6) cells. Cells transiently transfected with an empty vector were used as a knock-in control (OV-CTRL), while cells transfected with scramble siRNA represent the silencing control (Si-CTRL). The apoptosis status of RKO cells was first measured in the absence (WT) or presence of camptothecin (CAMPTO), a chemical compound that triggers apoptosis and, therefore, was used as a positive control. As expected, the camptothecin-treated cells showed a reduction in live cells from 90% to 60%, while the fraction of apoptotic cells increased from 10% to 40% compared to the WT control. Simultaneously, the fraction of dead cells remained unchanged. Furthermore, the apoptotic status of KI-CTRL and Si-CTRL (negative controls) was measured to check the impact of the transfection method on apoptosis. Although the transfection method used for silencing (Si-CTRL) resulted in a cell distribution similar to that of the WT control, the transfection used to overexpress NME6 (OV-CTRL) was stressful to the cells. In more detail, the overexpression diminished the number of live cells (from 90% to 45%) and caused the accumulation of apoptotic (from 10% to 35%) as well as dead cells (from 2% to 25%) when compared to the control (WT) (Figure S4).

**Figure 4:**
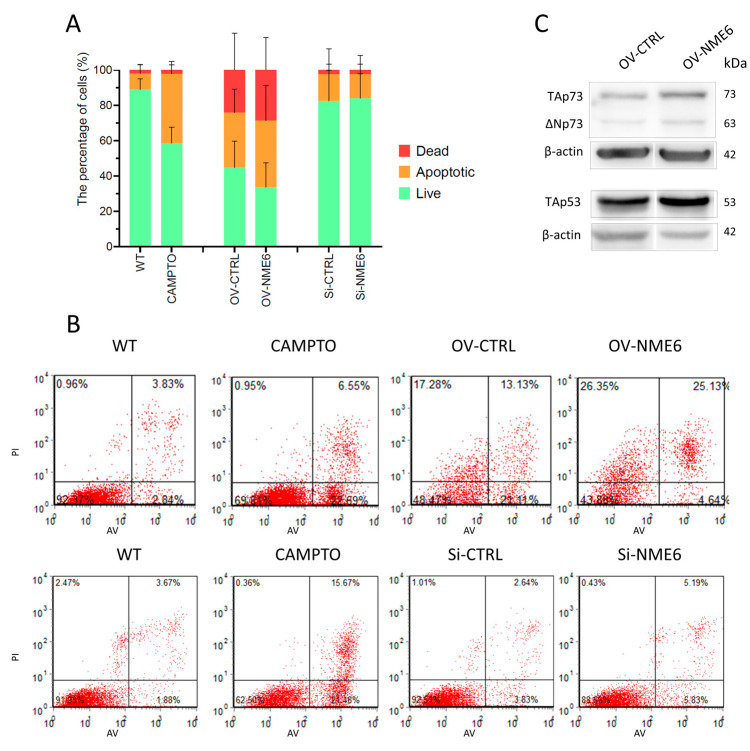
NME6 overexpression or silencing has no effect on apoptosis. (**A**) RKO cells (RKO), transfected with “empty” plasmid (OV-CTRL), transfected to overexpress NME6 (OV-NME6), and transfected with scramble siRNA (Si-CTRL) or with NME6 silencers (Si-NME6) were analyzed by flow cytometry after annexin V/Propidium iodide (AV/PI) staining. Live cells were negative for both markers (AV−/PI−). Apoptotic category includes both, early (AV+/PI−), and late (AV+/PI+) apoptotic cells. Remaining cells comprise the dead cell category (AV−/PI+). Data are expressed as mean ± SD (*n* = 4). (**B**) Representative flow cytometry scatter plots associated with (**A**). (**C**) The expression of p53 and p73 was analyzed on the protein level by Western blotting after transient NME6 overexpression in MDA-MB-231T cells (OV-NME6). Molecular weights of proteins are annotated on the right side of the membrane.

The analysis of apoptotic status after NME6 overexpression (OV-NME6) did not display differences when compared to an appropriate control (OV-CTRL) (Figure 4A,B). About 35 to 40% of cells were labeled as live, 35–40% were apoptotic, and 25–30% were dead cells. Similarly, the silencing of NME6 had no effect on apoptosis, with a very similar distribution of cells between Si-NME6 and the control (Si-CTRL) (80% live, 15% apoptotic, and 5% dead) (Figure 4A,B). As a result, the overexpression or silencing of NME6 in our experimental conditions seems to have no substantial impact on apoptosis in RKO cells. Nevertheless, these results should be considered cautiously regarding the NME6 overexpression experiment, given the fact that the transfection itself triggered massive apoptosis and cell death, which could potentially mask a minor effect of NME6 on apoptosis. However, in our experiments, NME6 does not appear to influence apoptosis.

Transcription factor p53 regulates cellular metabolism and modulates the balance between glycolysis and mitochondrial respiration. p53 can promote mitochondrial respiration in breast cancer cells [60,61], and when mutated, p53 is known to mediate the Warburg effect, thus promoting glycolysis [62,63]. Furthermore, mitochondrial metabolism can regulate p53 expression just as mitochondrial dysfunction can suppress p53 activity [64]. In addition, it has been shown that p53 family members have a significant impact on the cancer metabolic switch [60], while TAp73, an isoform of the p53 relative, was shown to regulate mitochondrial dynamics and the expression of OPA1 [65], a protein previously shown to be associated with NME6 [26]. Therefore, we analyzed whether NME6, as a potential regulator of mitochondrial respiration, influences p53 and p73 levels. We detected a moderate increase in both p53 and TAp73 levels in NME6-overexpressing MDA-MB-231T cells (OV-NME6) compared to the control (OV-CTRL) (Figure 4C and Figure S5). Based on our results, we can assume that NME6 can influence p53 and p73 expression; however, future studies are needed to elucidate the underlying molecular mechanisms of the NME6-dependent regulation of p53 and p73 expression.

NME6 is a mitochondrial protein closely related to energy production in the cell. Through its interaction with RCC1L, a protein affecting mitochondrial ribosome assembly and mitochondrial translation, NME6 overexpression was shown to impair mitochondrial respiration and decrease respiratory complexes’ abundance [26]. Further, NME6 was shown to actively participate in pyrimidine nucleotide salvage within the mitochondria, and its depletion resulted in decreased mitochondrial transcription, the destabilization of the respiratory chain, and a decrease in respiratory potential [27,28]. In light of these results, it seems obvious that an imbalance in NME6 expression negatively influences mitochondrial respiration and consequently affects the production of energy within the cell.

All the processes we investigated that were affected by the overexpression of NME6 directly or indirectly require a corresponding amount of energy [37,66,67,68], which is mainly produced in the mitochondria as ATP, the final product of mitochondrial respiration. Hence, the effect of NME6 overexpression on the cellular processes we observed in this study might be indirect and primarily caused by a shortage of energy due to the imbalanced levels of NME6 in mitochondria. However, testing this hypothesis is beyond the scope of this study. Indirect or not, the effect of NME6 expression on cell migration is undeniable and raises the question of the potential involvement of the protein in the metastatic cascade, since NME1, NME2, and the mitochondrial NME4 have already been described as metastasis suppressors [54,69]. Several in-depth studies are required to elucidate the involvement of NME6 in the metastatic spread.

## 3. Materials and Methods

### 3.1. Cells and Cell Maintenance

MDA-MB-231T cells (pleural effusion of breast adenocarcinoma) were donated by Dr. Patricia S. Steeg (Center for Cancer Research, National Cancer Institute, Bethesda, MD, USA), and RKO (human rectal carcinoma) cells were purchased from ATCC^®^ CCL-2™ (Manassas, VA, USA). The cells were grown in DMEM according to ATCC and the recommendations of our generous donors. They were supplemented with 10% fetal bovine serum (FBS), 1% streptomycin-penicillin, 1 mM sodium pyruvate, and 2 mM L-glutamine in a humidified chamber at 37 °C and 5% CO_2_. The MDA-MB-231T knock-in (KI) stable clones were prepared as described [26]. The cells were grown in humid atmosphere at 37 °C, with 5% CO_2_. Cell lines were tested to ensure they were mycoplasma-free.

### 3.2. Cell Cycle

MDA-MB-231T wild-type cells, stable clones with incorporated either “empty” plasmid (KI-CTRL) or NME6 encoding constructs (KI-NME6) were seeded in 6-well plates to reach 80% confluence on the day of the experiment. The cells were either allowed to grow for 72 h (KI experiment) or NME6 expression was silenced and analyzed 72 h afterwards (silencing experiment). On the day of the experiment, cells were harvested with trypsin. Pellet was washed in 1 mL PBS, fixed in 70% ethanol, and stored at 4 °C overnight. The next day, cells were resuspended in PBS supplemented with 0.1 µg/mL RNase. Cells were stained by adding propidium iodide at a final concentration of 40 µg/mL for 30 min at 37 °C in the dark. Analysis was performed using the BD FACSCalibur™ Flow Cytometer (BD Biosciences, Franklin Lakes, NJ, USA) or Muse Cell Analyzer (Merck Millipore, Burlington, MA, USA), while the data were analyzed using FlowJo software (v10.6.1, FlowJo, LLC, Ashland, OR, USA). Experiments were performed in triplicate. Data from related clones were pooled for statistical analysis.

### 3.3. Apoptosis

The apoptosis experiments were performed with RKO cells, as these have a p53 wild-type status. For this purpose, the self-prepared pcDNA3-NME6-186-FLAG plasmid and TurboFect transfection reagent (R0531, Thermo Scientific, Waltham, MA, USA) were used according to the manufacturer’s instructions. Cells were analyzed 48 h after transfection. Cells were silenced for NME6 using Dharmafect 4 transfection reagent (T-2004-02, Dharmacon, Lafayette, CO, USA) and NME6-silencers (L006755, Dharmacon) according to manufacturer’s instructions and analyzed 72 h post transfection. Scramble DNA and Scramble SiRNA were used as negative controls. Eighteen hours before the cells were harvested, the positive control for apoptosis was supplemented with camptothecin (2 µM final) directly in the medium, without replacing it, to induce apoptosis. On the day of the experiment, both the attached and the floating cells were detached using trypsin and collected. The pellet was resuspended in PBS, and 10^5^ cells were stained with the Annexin-V-FLUOS Staining Kit (11858777001, Roche, Basel, Switzerland) according to the manufacturer’s instructions. Analyses were performed using BD FACSCalibur™ Flow Cytometer (BD Biosciences, Franklin Lakes, NJ, USA), and the data were analyzed using FlowJo software (v10.6.1, FlowJo, LLC, Ashland, OR, USA). Experiments were performed in triplicate.

### 3.4. Wound-Healing Assay

For the wound-healing assay, 10^5^ cells were seeded in 24-well plates. Three KI-CTRL and three KI-NME6 stable clones were used for the knock-in experiment. For the knock-down experiment, cells were transiently silenced, and images were acquired 48 h (T = 0) and 72 h post silencing (T = 24 h). Cells were grown for 24 h to reach 90% confluence. To minimize the effect of proliferation, cells were starved for 24 h by replacing the medium with serum-free DMEM. The confluent cell layer was scratched with a pipette tip. The detached cells were washed with serum-free DMEM, photographed immediately (T = 0), and kept in the incubator for another 24 h in serum-free DMEM, after which a second image was taken (T = 24 h). The results were processed with ImageJ software (version 1.54f, Rasband, W.S., U. S. National Institutes of Health, Bethesda, MD, USA, https://imagej.nih.gov/ij/, (accessed on 10 October 2023)), using the plugin “MRI_Wound_Healing_Tool.ijm” to calculate the wound’s surface area at each time point (method: variance; variance filter radius: 20; threshold: 50; radius close: 4; and min. size: 10,000). The wound closure 24 h after scratching was calculated as (T0 area − T24 area)⁄(T0 area) and is expressed as a percentage. The data from related clones were pooled before statistical analysis.

### 3.5. Protein Extraction and Western Blotting

Proteins were extracted from cells in PBS supplemented with protease inhibitors (11836170001, Roche, Basel, Switzerland). The pellets were sonicated (2 × 10 s, 4 °C), and the protein concentration was determined using the BCA Protein Assay Kit (23227, Pierce, Woodland Hills, CA, USA).

Isolated protein extracts (30 µg) were separated on 10% or 12% SDS-PAGE, transferred to a nitrocellulose membrane, and immunoblotted for NME6 (Sigma Aldrich, St. Louis, MO, USA, HPA 017909, 1:1000), Fibronectin (Santa Cruz, Dallas, TX, USA, sc-8422, 1:200), E-cadherin (Santa Cruz, Dallas, TX, USA, sc-8426, 1:1000), N-cadherin (BD Biosciences, Franklin Lakes, NJ, USA, 610920, 1:500), β-catenin (Sigma Aldrich, St. Louis, MO, USA, C7207, 1:1000), Vimentin (Santa Cruz, Dallas, TX, USA, sc-32322, 1:1000), PCNA (Cell Signaling, Danvers, MA, USA, #13110, 1:1000), AKT (Cell Signaling, Danvers, MA, USA, #2920, 1:1000), pAKT (Cell Signaling, Danvers, MA, USA, #9271, 1:200), ERK1 (Santa Cruz, Dallas, TX, USA, sc-94, 1:1000), pERK (Cell Signaling, Dallas, TX, USA, #4377, 1:1000), Cyclin A (Santa Cruz, Dallas, TX, USA, sc-751, 1:500), Cyclin B (Santa Cruz, Dallas, TX, USA, sc-245, 1:500), Cyclin E (Santa Cruz, Dallas, TX, USA, sc-247, 1:400), p21 (Santa Cruz, Dallas, TX, USA, sc-397, 1:300), p27 (Santa Cruz, Dallas, TX, USA, sc-53871, 1:300), p73 (Abcam, Cambridge, UK, ab40658, 1:2000), or β-Actin (Sigma Aldrich, St. Louis, MO, USA, A3854, 1:10,000). Other antibodies, such as sheep pantropic anti-p53 antibody (clone SAPU, 1:5000), were kindly provided by J.C. Bourdon. The following HRP-linked secondary antibodies were used: anti-rabbit IgG (Cell signaling, Danvers, MA, USA, #7074, 1:5000), anti-mouse IgG (Cell signaling, Danvers, MA, USA, #7076, 1:5000), and anti-sheep IgG (Jackson ImmunoResearch Europe, Cambridge, UK, AB_2340709, 1:10,000). For protein visualization, we used Western Lightning Plus ECL Reagent (Perkin Elmer, Waltham, MA, USA, NEL104001EA) for most proteins or SuperSignal Western Blot Substrate Pico and Femto (3:1, 34096 and 34580, Thermo Scientific) for p53 and p73 on Alliance Q9 mini imaging system (UVitec, Cambridge, UK). When applicable, densitometry tests of the blots were performed using ImageJ software (version 1.54f, Rasband, W.S., U. S. National Institutes of Health, Bethesda, MD, USA, https://imagej.nih.gov/ij/, (accessed on 10 October 2023)), and the intensities of proteins of interest were normalized on beta-actin.

### 3.6. Statistical Analysis

Each experiment was performed independently two to four times. Statistical analysis was performed using GraphPad Prism (version 8.3.0) software. The statistical test used for the analysis is mentioned in the corresponding figure legend. Data are shown as the mean ± standard deviation (SD), and differences were considered statistically significant for a *p*-value < 0.05, which is marked by an asterisk (*).

## 4. Conclusions

In this work, we have shown that the overexpression of NME6 reduces cell migration and alters the EMT markers accordingly. Moreover, it influences the cell cycle upon DNA damage, affects the expression of proliferation markers, and impacts the MAPK/ERK signaling pathway. The observed changes in fundamental cellular processes might be a consequence of the energy imbalance caused by the overexpression of NME6 as already reported. However, extensive studies are still needed to decipher this interplay.

## Data Availability

Available from the corresponding author upon reasonable request.

## Supplementary Materials

The supporting information can be downloaded at: https://www.mdpi.com/article/10.3390/ijms25179580/s1.

## References

[1] Lascu I., Gonin P. (2000). The Catalytic Mechanism of Nucleoside Diphosphate Kinases. J. Bioenerg. Biomembr..

[2] Lacombe M.L., Milon L., Munier A., Mehus J.G., Lambeth D.O. (2000). The Human Nm23/Nucleoside Diphosphate Kinases. J. Bioenerg. Biomembr..

[3] Desvignes T., Pontarotti P., Fauvel C., Bobe J. (2009). Nme Protein Family Evolutionary History, a Vertebrate Perspective. BMC Evol. Biol..

[4] Gilles A.M., Presecan E., Vonica A., Lascu I. (1991). Nucleoside Diphosphate Kinase from Human Erythrocytes. Structural Characterization of the Two Polypeptide Chains Responsible for Heterogeneity of the Hexameric Enzyme. J. Biol. Chem..

[5] Steeg P.S., Bevilacqua G., Kopper L., Thorgeirsson U.P., Talmadge J.E., Liotta L.A., Sobel M.E. (1988). Evidence for a Novel Gene Associated With Low Tumor Metastatic Potential. JNCI J. Natl. Cancer Inst..

[6] Ćetković H., Harcet M., Roller M., Herak Bosnar M. (2018). A Survey of Metastasis Suppressors in Metazoa. Lab. Investig..

[7] Herak Bosnar M., Dubravčić K., Bago R., Pavelić J. (2008). Head and Neck Tumor Cells Exhibit Altered Proliferation upon Overexpression of Nm23 Genes. Croat. Chem. Acta.

[8] Ćetković H., Perina D., Harcet M., Mikoč A., Herak Bosnar M. (2015). Nme Family of Proteins—Clues from Simple Animals. Naunyn. Schmiedebergs. Arch. Pharmacol..

[9] Zhou X.-B., Feng Y.-X., Sun Q., Lukowski R., Qiu Y., Spiger K., Li Z., Ruth P., Korth M., Skolnik E.Y. (2015). Nucleoside Diphosphate Kinase B–Activated Intermediate Conductance Potassium Channels Are Critical for Neointima Formation in Mouse Carotid Arteries. Arterioscler. Thromb. Vasc. Biol..

[10] Cipollini G., Berti A., Fiore L., Rainaldi G., Basolo F., Merlo G., Bevilacqua G., Caligo M.A. (1997). Down-Regulation of the Nm23.H1 Gene Inhibits Cell Proliferation. Int. J. Cancer.

[11] Lombardi D., Lacombe M.-L., Paggi M.G. (2000). Nm23: Unraveling Its Biological Function in Cell Differentiation. J. Cell. Physiol..

[12] Bilitou A., Watson J., Gartner A., Ohnuma S. (2009). The NM23 Family in Development. Mol. Cell. Biochem..

[13] Lakso M., Steeg P.S., Westphal H. (1992). Embryonic Expression of Nm23 during Mouse Organogenesis. Cell Growth Differ..

[14] Boissan M., Montagnac G., Shen Q., Griparic L., Guitton J., Romao M., Sauvonnet N., Lagache T., Lascu I., Raposo G. (2014). Nucleoside Diphosphate Kinases Fuel Dynamin Superfamily Proteins with GTP for Membrane Remodeling. Science.

[15] Fournier H.-N., Albigès-Rizo C., Block M.R. (2003). New Insights into Nm23 Control of Cell Adhesion and Migration. J. Bioenerg. Biomembr..

[16] Li Y., Tong Y., Wong Y.H. (2015). Regulatory Functions of Nm23-H2 in Tumorigenesis: Insights from Biochemical to Clinical Perspectives. Naunyn. Schmiedebergs. Arch. Pharmacol..

[17] Feng Y., Gross S., Wolf N.M., Butenschön V.M., Qiu Y., Devraj K., Liebner S., Kroll J., Skolnik E.Y., Hammes H.-P. (2014). Nucleoside Diphosphate Kinase B Regulates Angiogenesis Through Modulation of Vascular Endothelial Growth Factor Receptor Type 2 and Endothelial Adherens Junction Proteins. Arterioscler. Thromb. Vasc. Biol..

[18] Chen C.-W., Wang H.-L., Huang C.-W., Huang C.-Y., Lim W.K., Tu I.-C., Koorapati A., Hsieh S.-T., Kan H.-W., Tzeng S.-R. (2019). Two Separate Functions of NME3 Critical for Cell Survival Underlie a Neurodegenerative Disorder. Proc. Natl. Acad. Sci. USA.

[19] Milon L., Rousseau-Merck M.F., Munier A., Erent M., Lascu L., Capeau J., Lacombe M.L. (1997). Nm23-H4, a New Member of the Family of Human Nm23/Nucleoside Diphosphate Kinase Genes Localised on Chromosome 16p13. Hum. Genet..

[20] Desvignes T., Pontarotti P., Bobe J. (2010). Nme Gene Family Evolutionary History Reveals Pre-Metazoan Origins and High Conservation between Humans and the Sea Anemone, Nematostella Vectensis. PLoS ONE.

[21] Mehus J.G., Deloukas P., Lambeth D.O. (1999). NME6: A New Member of the Nm23 /Nucleoside Diphosphate Kinase Gene Family Located on Human Chromosome 3p21.3. Hum. Genet..

[22] Proust B.L.J. (2022). Human NME6 Protein: Subcellular Localization, Structure and Function. Ph.D. Thesis.

[23] Boissan M., Schlattner U., Lacombe M.-L. (2018). The NDPK/NME Superfamily: State of the Art. Lab. Investig..

[24] Herak Bosnar M., Radić M., Ćetković H. (2018). A Young Researcher’s Guide to NME/Nm23/NDP Kinase. Period. Biol..

[25] Tsuiki H., Nitta M., Furuya A., Hanai N., Fujiwara T., Inagaki M., Kochi M., Ushio Y., Saya H., Nakamura H. (2000). A Novel Human Nucleoside Diphosphate (NDP) Kinase, Nm23-H6, Localizes in Mitochondria and Affects Cytokinesis. J. Cell. Biochem..

[26] Proust B., Radić M., Vidaček N.Š., Cottet C., Attia S., Lamarche F., Ačkar L., Mikulčić V.G., Tokarska-Schlattner M., Ćetković H. (2021). NME6 Is a Phosphotransfer-Inactive, Monomeric NME/NDPK Family Member and Functions in Complexes at the Interface of Mitochondrial Inner Membrane and Matrix. Cell Biosci..

[27] Grotehans N., McGarry L., Nolte H., Xavier V., Kroker M., Narbona-Pérez Á.J., Deshwal S., Giavalisco P., Langer T., MacVicar T. (2023). Ribonucleotide Synthesis by NME6 Fuels Mitochondrial Gene Expression. EMBO J..

[28] Kramer N.J., Prakash G., Isaac R.S., Choquet K., Soto I., Petrova B., Merens H.E., Kanarek N., Churchman L.S. (2023). Regulators of Mitonuclear Balance Link Mitochondrial Metabolism to MtDNA Expression. Nat. Cell Biol..

[29] Seifert M., Welter C., Mehraein Y., Seitz G. (2005). Expression of the Nm23 Homologues Nm23-H4, Nm23-H6, and Nm23-H7 in Human Gastric and Colon Cancer. J. Pathol..

[30] Ke J., Lou J., Zhong R., Chen X., Li J., Liu C., Gong Y., Yang Y., Zhu Y., Zhang Y. (2016). Identification of a Potential Regulatory Variant for Colorectal Cancer Risk Mapping to 3p21.31 in Chinese Population. Sci. Rep..

[31] Wang C.-H., Ma N., Lin Y.-T., Wu C.-C., Hsiao M., Lu F.L., Yu C.-C., Chen S.-Y., Lu J. (2012). A ShRNA Functional Screen Reveals Nme6 and Nme7 Are Crucial for Embryonic Stem Cell Renewal. Stem Cells.

[32] Ernst O., Sun J., Lin B., Banoth B., Dorrington M.G., Liang J., Schwarz B., Stromberg K.A., Katz S., Vayttaden S.J. (2021). A Genome-Wide Screen Uncovers Multiple Roles for Mitochondrial Nucleoside Diphosphate Kinase D in Inflammasome Activation. Sci. Signal..

[33] Vyas S., Zaganjor E., Haigis M.C. (2016). Mitochondria and Cancer. Cell.

[34] Lossaint G., Horvat A., Gire V., Bačević K., Mrouj K., Charrier-Savournin F., Georget V., Fisher D., Dulić V. (2022). Reciprocal Regulation of P21 and Chk1 Controls the Cyclin D1-RB Pathway to Mediate Senescence Onset after G2 Arrest. J. Cell Sci..

[35] Diller L., Kassel J., Nelson C.E., Gryka M.A., Litwak G., Gebhardt M., Bressac B., Ozturk M., Baker S.J., Vogelstein B. (1990). P53 Functions as a Cell Cycle Control Protein in Osteosarcomas. Mol. Cell. Biol..

[36] Pruteanu L.L., Braicu C., Módos D., Jurj M.A., Raduly L.Z., Zănoagă O., Magdo L., Cojocneanu R., Paşca S., Moldovan C. (2022). Targeting Cell Death Mechanism Specifically in Triple Negative Breast Cancer Cell Lines. Int. J. Mol. Sci..

[37] Salazar-Roa M., Malumbres M. (2017). Fueling the Cell Division Cycle. Trends Cell Biol..

[38] Sakamaki T., Casimiro M.C., Ju X., Quong A.A., Katiyar S., Liu M., Jiao X., Li A., Zhang X., Lu Y. (2006). Cyclin D1 Determines Mitochondrial Function InVivo. Mol. Cell. Biol..

[39] Taguchi N., Ishihara N., Jofuku A., Oka T., Mihara K. (2007). Mitotic Phosphorylation of Dynamin-Related GTPase Drp1 Participates in Mitochondrial Fission. J. Biol. Chem..

[40] Mitra K., Wunder C., Roysam B., Lin G., Lippincott-Schwartz J. (2009). A Hyperfused Mitochondrial State Achieved at G1-S Regulates Cyclin E Buildup and Entry into S Phase. Proc. Natl. Acad. Sci. USA.

[41] Wang Z., Fan M., Candas D., Zhang T.Q., Qin L., Eldridge A., Wachsmann-Hogiu S., Ahmed K.M., Chromy B.A., Nantajit D. (2014). Cyclin B1/Cdk1 Coordinates Mitochondrial Respiration for Cell-Cycle G2/M Progression. Dev. Cell.

[42] Rosenthal C.K. (2014). Cdk1 Boosts Mitochondrial Energy Production. Nat. Cell Biol..

[43] Poikonen P., Sjöström J., Amini R.M., Villman K., Ahlgren J., Blomqvist C. (2005). Cyclin A as a Marker for Prognosis and Chemotherapy Response in Advanced Breast Cancer. Br. J. Cancer.

[44] Dutta A., Chandra R., Leiter L.M., Lester S. (1995). Cyclins as Markers of Tumor Proliferation: Immunocytochemical Studies in Breast Cancer. Proc. Natl. Acad. Sci. USA.

[45] Zheng S., Liu T., Liu Q., Yang L., Zhang Q., Han X., Shen T., Zhang X., Lu X. (2020). Widely Targeted Metabolomic Analyses Unveil the Metabolic Variations after Stable Knock-down of NME4 in Esophageal Squamous Cell Carcinoma Cells. Mol. Cell. Biochem..

[46] Wang W., Dong M., Cui J., Xu F., Yan C., Ma C., Yi L., Tang W., Dong J., Wei Y. (2019). NME4 May Enhance Non-small Cell Lung Cancer Progression by Overcoming Cell Cycle Arrest and Promoting Cellular Proliferation. Mol. Med. Rep..

[47] Zheng S., Liu Q., Liu T., Yang L., Zhang Q., Shen T., Zhang X., Han X., Lu X. (2020). NME4 Modulates PD-L1 Expression via the STAT3 Signaling Pathway in Squamous Cell Carcinoma. Biochem. Biophys. Res. Commun..

[48] Olaisen C., Müller R., Nedal A., Otterlei M. (2015). PCNA-Interacting Peptides Reduce Akt Phosphorylation and TLR-Mediated Cytokine Secretion Suggesting a Role of PCNA in Cellular Signaling. Cell. Signal..

[49] Paplomata E., O’regan R. (2014). The PI3K/AKT/MTOR Pathway in Breast Cancer: Targets, Trials and Biomarkers. Ther. Adv. Med. Oncol..

[50] Whyte J., Bergin O., Bianchi A., McNally S., Martin F. (2009). Key Signalling Nodes in Mammary Gland Development and Cancer. Mitogen-Activated Protein Kinase Signalling in Experimental Models of Breast Cancer Progression and in Mammary Gland Development. Breast Cancer Res..

[51] Ortega M.A., Fraile-Martínez O., Asúnsolo Á., Buján J., García-Honduvilla N., Coca S. (2020). Signal Transduction Pathways in Breast Cancer: The Important Role of PI3K/Akt/MTOR. J. Oncol..

[52] Xu W., Yang Z., Lu N. (2015). A New Role for the PI3K/Akt Signaling Pathway in the Epithelial-Mesenchymal Transition. Cell Adhes. Migr..

[53] Olea-Flores M., Zuñiga-Eulogio M.D., Mendoza-Catalán M.A., Rodríguez-Ruiz H.A., Castañeda-Saucedo E., Ortuño-Pineda C., Padilla-Benavides T., Navarro-Tito N. (2019). Extracellular-Signal Regulated Kinase: A Central Molecule Driving Epithelial–Mesenchymal Transition in Cancer. Int. J. Mol. Sci..

[54] Lacombe M.-L., Lamarche F., De Wever O., Padilla-Benavides T., Carlson A., Khan I., Huna A., Vacher S., Calmel C., Desbourdes C. (2021). The Mitochondrially-Localized Nucleoside Diphosphate Kinase D (NME4) Is a Novel Metastasis Suppressor. BMC Biol..

[55] Jonkman J.E.N., Cathcart J.A., Xu F., Bartolini M.E., Amon J.E., Stevens K.M., Colarusso P. (2014). Cell Adhesion & Migration An Introduction to the Wound Healing Assay Using Livecell Microscopy An Introduction to the Wound Healing Assay Using Livecell Microscopy. Cell Adhes. Migr..

[56] Zanotelli M.R., Goldblatt Z.E., Miller J.P., Bordeleau F., Li J., VanderBurgh J.A., Lampi M.C., King M.R., Reinhart-King C.A. (2018). Regulation of ATP Utilization during Metastatic Cell Migration by Collagen Architecture. Mol. Biol. Cell.

[57] Loh C.-Y., Chai J.Y., Tang T.F., Wong W.F., Sethi G., Shanmugam M.K., Chong P.P., Looi C.Y. (2019). The E-Cadherin and N-Cadherin Switch in Epithelial-to-Mesenchymal Transition: Signaling, Therapeutic Implications, and Challenges. Cells.

[58] Haerinck J., Berx G. (2021). Partial EMT Takes the Lead in Cancer Metastasis. Dev. Cell.

[59] Bae Y.-H., Shin J.-M., Park H.-J., Jang H.-O., Bae M.-K., Bae S.-K. (2014). Gain-of-Function Mutant P53-R280K Mediates Survival of Breast Cancer Cells. Genes Genom..

[60] Moulder D.E., Hatoum D., Tay E., Lin Y., McGowan E.M. (2018). The Roles of P53 in Mitochondrial Dynamics and Cancer Metabolism: The Pendulum between Survival and Death in Breast Cancer?. Cancers.

[61] Matoba S., Kang J.-G., Patino W.D., Wragg A., Boehm M., Gavrilova O., Hurley P.J., Bunz F., Hwang P.M. (2006). P53 Regulates Mitochondrial Respiration. Science.

[62] Zhang C., Liu J., Liang Y., Wu R., Zhao Y., Hong X., Lin M., Yu H., Liu L., Levine A.J. (2013). Tumour-Associated Mutant P53 Drives the Warburg Effect. Nat. Commun..

[63] Ma W., Sung H.J., Park J.Y., Matoba S., Hwang P.M. (2007). A Pivotal Role for P53: Balancing Aerobic Respiration and Glycolysis. J. Bioenerg. Biomembr..

[64] Compton S., Kim C., Griner N.B., Potluri P., Scheffler I.E., Sen S., Jerry D.J., Schneider S., Yadava N. (2011). Mitochondrial Dysfunction Impairs Tumor Suppressor P53 Expression/Function. J. Biol. Chem..

[65] Buckley N., Craxton A., Sun X.-M., Panatta E., Pinon L., Llodrá J., Morone N., Amelio I., Melino G., Martins L.M. (2023). TAp73 Regulates Mitochondrial Dynamics and Multiciliated Cell Homeostasis through an OPA1 Axis. bioRxiv.

[66] Li Y., Yao L., Mori Y., Sun S.X. (2019). On the Energy Efficiency of Cell Migration in Diverse Physical Environments. Proc. Natl. Acad. Sci. USA.

[67] Zhu J., Thompson C.B. (2019). Metabolic Regulation of Cell Growth and Proliferation. Nat. Rev. Mol. Cell Biol..

[68] Ortega-Arzola E., Higgins P.M., Cockell C.S. (2024). The Minimum Energy Required to Build a Cell. Sci. Rep..

[69] Prunier C., Chavrier P., Boissan M. (2023). Mechanisms of Action of NME Metastasis Suppressors—A Family Affair. Cancer Metastasis Rev..

