# Supplementary material for: Mitochondrial NME6 Influences Basic Cellular Processes in Tumor Cells In Vitro
Source: Int J Mol Sci. 2024 Sep 4;25(17):9580. doi: 10.3390/ijms25179580 (PMC11395177; doi:10.3390/ijms25179580)
Supplement: Supplementary file 1 [file ijms-25-09580-s001.zip › ijms-3075982-supplementary.pdf]

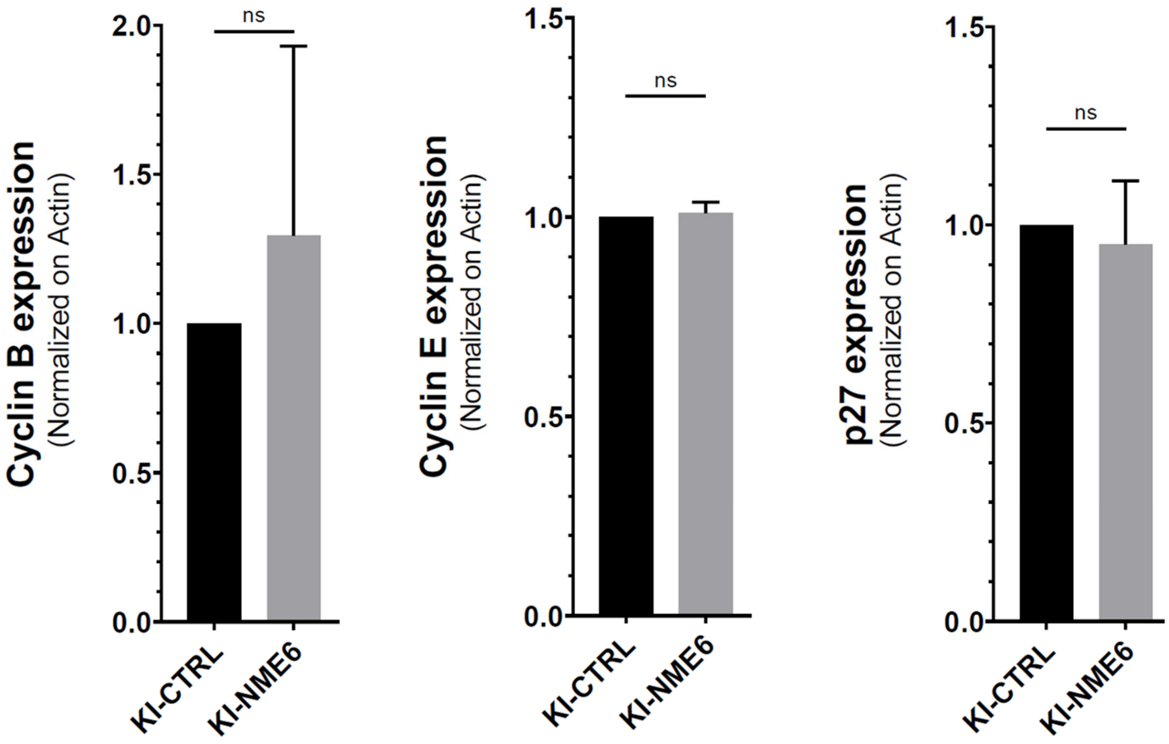

Figure S1. The levels of cyclin B, cyclin E and p27 shown in Figure 1 G were analyzed by blot densitometry. “ns” indicates not significant ($p > 0.05$) (Mean ± SD, Unpaired t-test; n=3).

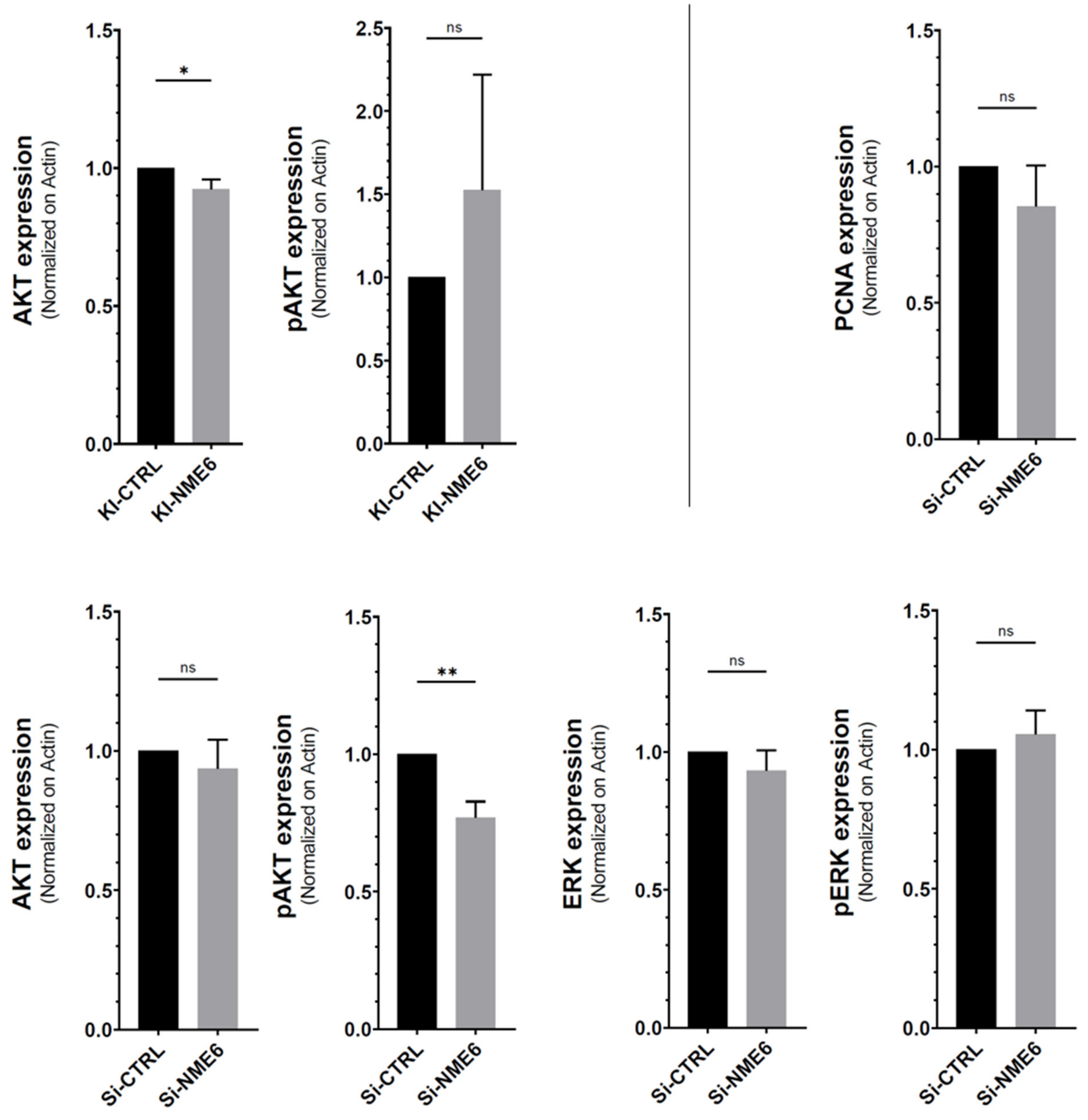

Figure S2. The levels of PCNA, ERK and pERK as well as AKT and pAKT shown in Figures 2 A and 2 C were analyzed by blot densitometry. Significance is shown as ** p< 0.01; * p<0.05, whereas "ns" indicates not significant (p> 0.05) (Mean ± SD, Unpaired t-test; n=3).

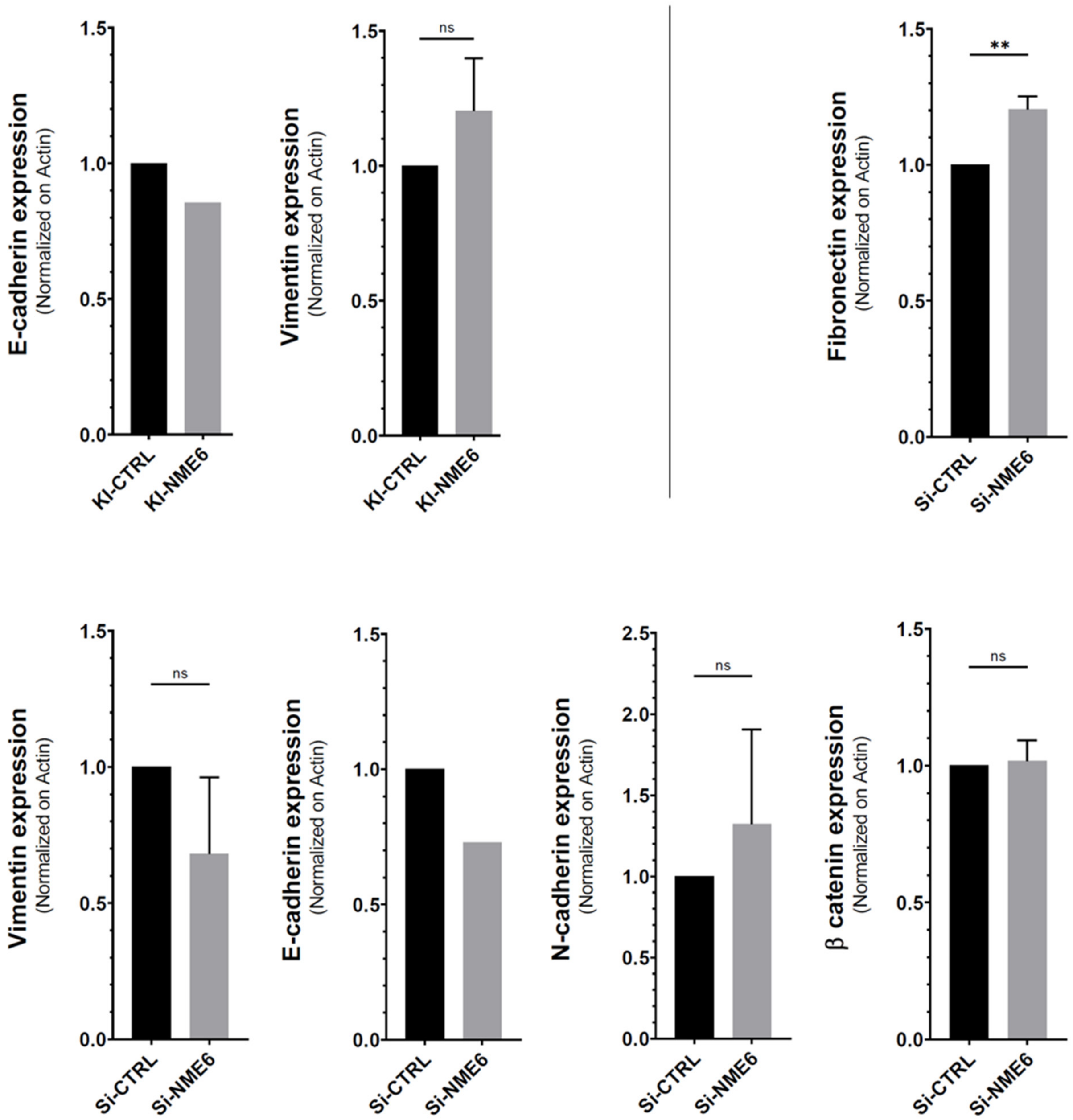

Figure S3. The levels of fibronectin, E-cadherin, N-cadherin, β-catenin and vimentin shown in Figures 3 B and 3 E were analyzed by blot densitometry. Significance is shown as ** $p < 0.01$; whereas "ns" indicates not significant ($p > 0.05$) (Mean ± SD, Unpaired t-test; n=3).

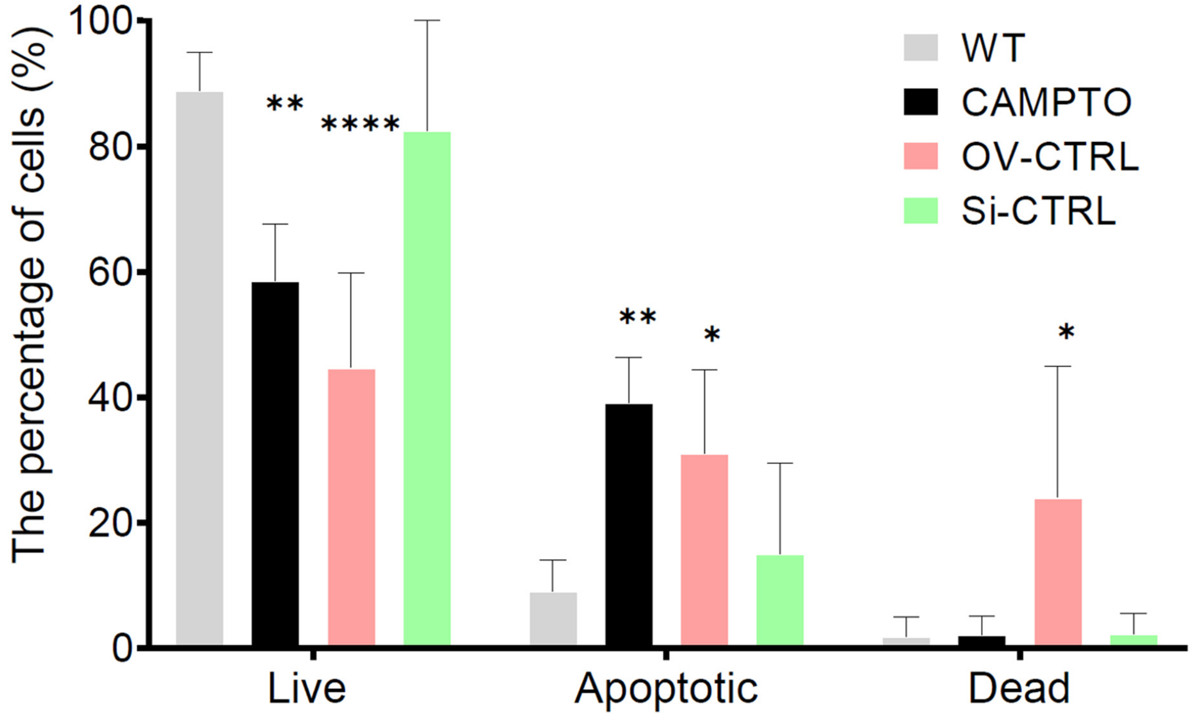

Figure S4. Transfection reagents can trigger apoptosis and cell death. RKO cells (WT), transfected with empty plasmid as a negative control for KI (OV-CTRL), or transfected with scramble siRNA as a negative control for silencing (Si-CTRL) were assayed by flow cytometry after annexin V/Propidium iodide (AV/PI) staining. RKO cells treated with camptothecin (CAMPTO) were used as a positive control. Live cells were negative for both markers (AV-/PI-). Apoptotic category regroups both the early (AV+/PI-) and late (AV+/PI+) apoptotic cells. Remaining cells compose the dead cell category (AV-/PI+). Data are given as mean ± SD (n=4). For comparison between RKO and other conditions, significance is given as **** $p<0.0001$; ** $p< 0.01$; * $p< 0.05$ (Two-way ANOVA).

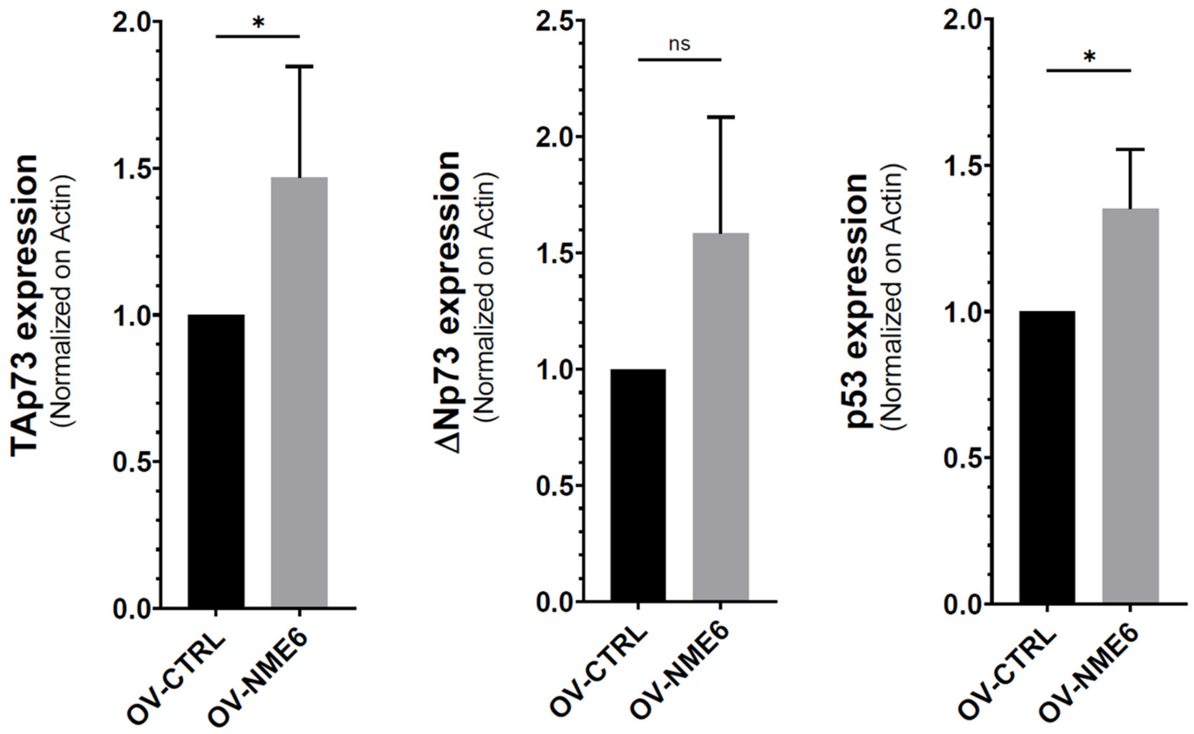

Figure S5. The levels of p53, TAp73 and ΔNp73 shown in Figure 4 C were analyzed by blot densitometry. Significance is shown as * $p<0.05$, whereas "ns" indicates not significant ($p>0.05$) (Mean ± SD, Unpaired t-test; n=3).
